# Medical Opinion and Movement

**Published:** 1910-02-19

**Authors:** 


### Medical Opinion and Movement.

### Effect of Drugs on Blood-vessels.

Drs. Eppinger and Hess have studied, according to the method of E. O. Meyer, the action of certain drugs that are known to influence in some way the vascular system. Blood-vessels removed from animals immediately after death are suspended in a solution containing the drug to be studied, and the changes in their length which they undergo are registered and observed by means of a kymograph. Comparing the effects produced on peripheral arteries with those on the coronary arteries it is found that these two kinds of vessels are affected differently by some of the drugs. Thus with adrenalin the coronary arteries dilate, while the peripheral arteries contract. Just the inverse happens with choline, eserine, and pilocarpine. The digitalis group cause contraction of both kinds of vessels, and atropine, ergotine, and the nitrites cause dilatation. Also caffeine and theobromine dilate the two kinds of vessels. Iodides are found to have no effect at all. The authors suggest that these facts are of therapeutic interest, for in some cases it would be desirable to associate two drugs together in order to produce an effect at the same time upon the vessels of the heart and on those of the periphery.

### Wassermann's Reaction in Milk.

From the researches of O. Thomsen made at the Copenhagen Institute of Serotherapy, and reported in the *Berliner Klinische Wochenschifte*, it would appear that the milk of syphilitic women often gives Wassermann's reaction, even in cases where the blood serum has failed to give a positive result. The reaction shows itself in the milk, two or three days before childbirth. If the woman suckles her child the intensity of the reaction rapidly disappears, until, in five or six days after parturition, it can no longer be found. If, on the other hand, the woman does not nurse her child, the reaction continues as long as there is milk to be found, a matter of two or three weeks. The milk of non-syphilitic women may for a few days after childbirth give the reaction, though this is much less marked than in the syphilitic cases. It would be premature at the present moment to judge of the diagnostic and prognostic value of local reactions such as those given by the milk and the cerebro-spinal fluid. The author is, nevertheless, convinced that there is an intimate connection between reactions obtained from such sources and that given by the blood serum.

### Balsam of Peru as a Nasal Antiseptic.

Dr. Henri Bourgeois, writing in *Le Progrès Médical*, points out that a proper disinfection of the nasal fossæ and naso-pharynx of individuals exposed to influenza infection or suffering from influenza, scarlatina, measles, and the like would constitute a valuable prophylactic measure. In the infectious fevers many complications—otitis, mastoiditis, broncho-pneumonia, etc., might be saved by such a measure. Nasal irrigations are entirely abandoned in the course of acute infections on account of the many cases of otitis which have followed their use. Vapour inhalations of menthol are of some service by their vaso-constricting effects, but their antiseptic action is little or nil. On the other hand, the author is of opinion that the nebular spray is almost useless, and that its effect does not extend beyond the anterior part of the nasal fossæ. He advocates the use of Balsam of Peru made up into an ointment, which can be sniffed up both nostrils into the naso-pharynx. The formula for the ointment is: Balsam of Peru 0.75 grammes, lanoline 5 grammes, and vaseline 10 grammes. The ointment should be put up in a metal tube. The patient should lie down and place the mouth of the tube in the nostril and press the ointment out, and at the same time draw it up with repeated sniffs, till he feels it in the naso-pharynx. The excess of ointment in the naso-pharynx may then be expectorated. The author is of opinion that such an application night and morning may prevent the development of a coryza, and should be used as a prophylactic measure by individuals liable to catarrh in winter or by those in the midst of an influenza infection. He also advocates its use, as mentioned above, in cases of the infectious fevers to prevent naso-pharyngeal complications.

### Bacteriology of Measles.

Dr. Paul Sittler has been investigating the morbid agent of measles, and an account of his work appears in the *Münchener Medizinische Wochenschrift*. Cultures taken from the naso-pharynx of patients suffering from measles show a variety of microbes, but among them Dr. Sittler has found an organism closely similar to the staphylococcus albus, which always predominates. Examinations carried out in a similar manner in cases of scarlatina, German measles, varicella, and diphtheria have failed to show the same predominance of this microbe. It would appear, therefore, to be a special factor in the pathology of measles and not to form one of the common agents of a superadded infection. In order to estimate its specificity Sittler submitted it to the test of agglutination by blood serum. He finds that the serum of children who have had measles agglutinates the microbe in question strongly, whereas the serum of those who have not suffered from the disease does not agglutinate it. In regard to closely allied staphylococci taken from cases of German measles, the serum of the same children who have had measles only showed a very feeble agglutinative power. Sittler has, moreover, developed a somewhat ingenious hypothesis in regard to the origin of the exanthem of measles. He suggests that the cause of the disease consists in the infection of the upper respiratory passages by this staphylococcus, that the microbe develops more or less rapidly and sets at liberty certain soluble toxins. These toxins are absorbed into the system and determine the production of anti-bodies. The exanthem is the result of this production of anti-bodies by a mechanism similar to that which causes the appearance of a serum rash. In other words, the rash of measles is an anaphylactic reaction of the organism to the toxins of this specific microbe, similar to the anaphylactic reaction of the organism to injected serums. The other manifestations of the disease, on the part of the mucous membranes, are of a similar nature, and on these inflamed surfaces the specific microbe has every facility to proliferate. Hence the extreme infectivity of the disease at this period. In scarlatina, on the other hand, the author ascribes the exanthem to the direct action of the streptococcic toxins.

### Corpus Luteum and the Menopause.

Morley, in the *Journal of the Michigan State Medical Society*, reports his results in the treatment of disturbances of the artificial and physiological menopause by extract of corpus luteum. The extract used was obtained from the corpora lutea of cows' ovaries. Since the consensus of opinion seems to be that the internal secretion of the ovary is produced by the yellow bodies, the author chose an extract made from these bodies rather than one obtained from the whole organ. The extract was given in five-grain doses three times a day thirty minutes to one hour after meals. The results in eighteen cases were as follows: Five patients were cured, twelve improved, and one obtained no relief. Of these eighteen cases fourteen suffered from disturbances due to artificial or operative menopause. In the remaining four cases the disturbances were the result of a natural menopause. While not wishing to draw definite conclusions from such a small series of cases, the author considers his results sufficiently favourable to justify further treatment on similar lines in cases of the same nature.

### Exostosis on the Calcaneum.

Reclus and Schwartz in the *Rèvue de Chirurgie* give an account of the varieties of exostosis met with on the calcaneum. These are of two kinds, the sub-calcanean and the retro-calcanean. The exostosis develops slowly and insidiously as the result either of trauma or of urethritis. In the first or sub-calcanean variety the bony prominence forms under the heel in the neighbourhood of the internal tuberosity. The patient walks on tip-toe and complains of pain in the heel in so doing, which, however, disappears in the recumbent position. In the second variety the exostosis makes its appearance at the posterior surface of the calcaneum in its upper part immediately in front of the tendo Achillis, where lies the serous bursa. A chronic hygroma of this bursa is found at the same time. The patient suffers while walking, especially when bringing the triceps into play. Clinically the tendo Achillis is found thickened, especially along its borders and in the latero-tendinous gutter.

### Salol in Tooth-powder.

The dangers of using tooth-powder in which salol is a constituent are drawn attention to in an article in *Le Lyon Médical*, as the result of an observation of Dubreuil, who states that he has met with several cases of labial eczema in persons who have used powder containing this drug. The decomposition of salol into salicylic acid and phenol sets up an irritation of the labio-buccal mucous membrane and of the skin of the lips. The writer goes on to remark that having regard to the frequent presence of the drug in tooth-powders and to the comparative rarity of labial eczema in the users thereof, the risks of contracting the condition cannot be very great.

### Extraction of the Cataractous Lens.

The Extraction of the Cataractous Lens in its Capsule, commonly known in India as Smith's operation, has not found very much favour with ophthalmologists. Thus Major Herbert is strongly opposed to it, and Major Birdwood published in 1906 311 cases in which he had tried the method with results which were decidedly unfavourable. The latter expert, however, has now had an opportunity of learning the exact technique from Major Smith at Jullundur, and is satisfied that his previous method, based merely on Maynard's and Smith's descriptions of the operation, was quite different from the proper technique of the operation as practised by its inventor. Moreover, with his corrected style learnt at Jullundur, he is able to get results similar to those claimed by Smith; and he believes that this operation is greatly superior to the old extra-capsular one. The full description of the operation, which requires very delicate manipulation, is given by Major Birdwood in the *Indian Medical Gazette*. The operator must be sitting down on a stool behind the patient's head. This is important as it gives the operator great steadiness of hand compared with that obtainable in the standing position. The eyebrows, lids, and instruments are sterilised in whatever method each operator prefers. A speculum is inserted between the lids. The eyebrow is then strongly drawn upwards, and the speculum also raised off the globe. A stream of 1 in 2,000 perchloride of mercury is then strongly douched into the fornices from an irrigator three feet above the patient's head. By drawing the eyebrow upwards every corner of the upper fornix is made visible and easily reached by the lotion.

The incision is then made. It must be very nearly but not quite half the circumference of the cornea. It is commenced slightly behind the sclerocorneal junction and brought out slightly in the cornea, the edge of the knife being turned a little upwards before completion of the incision. An iridectomy of moderate size is then done. The speculum is removed, and all fluid is squeezed out of the conjunctival sac by pressing a piece of cotton wool across the closed lids from the inner to the outer canthus. The assistant then takes a stout strabismus hook in the thumb and index finger of his right hand and draws the upper lid vertically forwards. The operator then cannot see the globe unless he leans over the right shoulder of the patient. At the same time with the middle, ring and small finger of the same hand the assistant draws the eyebrow forcibly up. In this way there is no pressure whatever on the globe. With the thumb of his left hand he draws the lower lid down. Smith attaches great importance to the method in which the assistant elevates the lid. It is a little difficult at first, but after a few days an intelligent assistant can be taught it. The operator then leans well over the right shoulder of the patient and looks at the globe over the cheek. He takes a strabismus hook in his right hand and places the point on the cornea over the point where he thinks the lower edge of the lens is. Pressure is made at first downwards and then from side to side and backwards and forwards; the downward pressure is kept up gently till the edge of the lens appears in the gaping wound. The spoon, sometimes held in the left hand, is placed on the cornea beside the hook and used to steady the lens and keep up the tension at one side, while the point of the hook increases the tension at the other side; as the lens emerges the point of the hook follows it up.

### Functions of the Semilunar Ganglia.

The following is a short account of the case which led Exner and Jaeger to undertake experiments with a view to determining the functions of the semilunar ganglia. A man of forty had for three years suffered from epigastric pains and constipation. Owing to the existence of visible peristaltic contractions of the small intestine, a diagnosis of stenosis was made. Laparotomy, however, showed no trace of this stenosis, but at certain points in the course both of the large and small intestine, marked contractions of the intestines were noticed. At the same time an indurated mass was noticed in the middle line above the pancreas and behind the peritoneum. Thereupon the abdomen was closed without further intervention. No improvement followed this operation, and in a short time symptoms of pyloric stenosis necessitated the performance of a gastro-enterostomy. At this second operation an adherent tumour the size of an apple was found behind the pylorus. This was considered to be a carcinoma. The patient died three months later, and at the autopsy a cicatricial stenosis of both the cardiac and pyloric ends of the stomach was found, with inflammatory induration involving the retro-peritoneal tissue above, and surrounding the pancreas. The retro-peritoneal nerves were, unfortunately, not dissected out; but in spite of this lacuna in the anatomical examination, the authors attribute the troubles in intestinal motility, as found both clinically and at the first operation, to the compression of the cœliac plexus by the retro-peritoneal mass. To establish this hypothesis they undertook a series of experiments on cats, with the object of elucidating the action of the semilunar ganglia on intestinal movements. They found that after removal of these ganglia, the contractions of the intestines induced by direct electrical stimulation are longer and more energetic than before removal. The ganglia are inhibitory. In cases of intestinal spasm of doubtful origin, the possibility of this being due to some alteration in the ganglia must not be lost sight of.

### Electricity in the Treatment of Tabes.

In the *Deutsche Med. Woch.*, M. Herzog reports the results he has obtained by using Vesical Faradisation in the treatment of cases of Tabes Dorsalis complicated by Cystitis and Pyelonephritis. He introduces one electrode into the urethra to a depth of one centimetre, the other being placed on the lumbar spine. The current is then allowed to pass for three minutes. The author has treated four cases in this way. One patient was improved from the very first application, not only in his vesical condition, but in his power of locomotion. This improvement increased, and at the end of the thirtieth application the patient was able to resume his military duties, his ataxy being unnoticeable except to the trained eye. Equally good results were obtained in two of the other cases; but in the last the result, though satisfactory, was less favourable. The author explains the efficacy of this treatment by the transmission to the spinal cord, along those sensory paths which are still intact, of vesical stimuli induced by the faradic current.

### Retention of Urine.

An interesting case of Retention of Urine after eating sorrel is mentioned by Professor Carrière in *Le Journal de Méd. et de Chir. Prat.* When seen, the patient, a girl of six, had experienced difficulty in micturition for three or four days, severe straining for twenty minutes or so being necessary before the bladder could be emptied. Nothing either in the local or general condition of the patient could be found to account for these symptoms. On analysing the urine, it was found to be excessively acid, this being due to oxalic acid in the form of calcium oxalate, which was present in five times the normal amount. The patient had eaten a considerable quantity of sorrel leaves before the onset of symptoms. She was placed on a milk diet, with small doses of sodium bicarbonate, and in two days the difficulty in micturition ceased. Four days later the oxaluria disappeared. The author also mentions another case of the same nature in a boy of five, who, after eating sorrel in quantity, suffered with retention of urine for thirty-two hours, necessitating the use of a catheter. In this case again milk diet and sodium bicarbonate effected a cure. Such cases are uncommon, and Professor Carrière's report is an interesting contribution to the literature of toxicology. Sorrel, however, contains other things besides oxalates, and it would be interesting to find if these had anything to do with the retention.

